# Dual use of VA and non-VA hospitals by Veterans with multiple hospitalizations

**DOI:** 10.1186/s12913-015-1069-8

**Published:** 2015-09-29

**Authors:** Alan N. West, Mary E. Charlton, Mary Vaughan-Sarrazin

**Affiliations:** Research Service, VA Medical Center (10A5A), 215 N. Main St., White River Junction, Vermont, 05009 USA; Geisel Medical School (formerly Dartmouth Medical School), Hanover, New Hampshire USA; Veterans Rural Health Resource Center – Eastern Region, White River Junction, Vermont, USA; Iowa City VA Health Care System, Comprehensive Access and Delivery Research and Evaluation (CADRE) Center, Iowa City, Iowa USA; Department of Epidemiology, The University of Iowa College of Public Health, Iowa City, Iowa USA; Department of Internal Medicine, The University of Iowa Carver College of Medicine, Iowa City, Iowa USA

**Keywords:** Veterans, Non-VA hospitalizations, Dual use, Psychiatry, Insurance

## Abstract

**Background:**

Veterans who are hospitalized in both VA and non-VA hospitals within a short timespan may be at risk for fragmented or conflicting care. To determine the characteristics of these “dual users,” we analyzed administrative hospital discharge data for VA-enrolled veterans of any age in seven states, including any VA or non-VA hospitalizations they had in 2004–2007.

**Method:**

For VA enrollees in Arizona, Iowa, Louisiana, Florida, South Carolina, Pennsylvania, or New York in 2007, we merged 2004–2007 discharge data for all VA hospitalizations and all non-VA hospitalizations listed in state health department or hospital association databases. For patients hospitalized in 2007, we compared those younger or older than 65 years who had one or multiple hospitalizations during the year, split into users of VA hospitals, non-VA hospitals, or both (“dual users”), on demographics, priority for VA care, travel times, principal diagnoses, co-morbidities, lengths of stay, and prior (2004–2006) hospitalizations, using chi-square analysis or ANOVA. Multiply hospitalized patients were compared with multinomial logistic regressions to predict non-VA and dual use. Payers for non-VA hospitalizations also were compared across groups.

**Results:**

Of unique inpatients in 2007, 38 % of those 65 or older were hospitalized more than once during the year, as were 32 % of younger patients; 3 and 8 %, respectively, were dual users. Dual users averaged the most index-year (3.7) and prior (1.5) hospitalizations, split evenly between VA and non-VA. They also had higher rates of admission for circulatory diseases, symptoms/signs/ill-defined conditions, and injury and poisoning, and more admissions for multiple diagnostic categories; among younger patients they had the highest rate of mental disorders admissions. Higher income, non-rural residence, greater time to VA care, lower VA priority, prior non-VA hospitalization, no prior VA hospitalization, and several medical categories predicted greater non-VA use. Among younger patients, however, mental disorders predicted more dual use but less exclusively non-VA use. Dual users’ non-VA admissions were more likely than others’ to be covered by payers other than Medicare or commercial insurance.

**Conclusions:**

Younger dual users require more medical and psychiatric treatment, and rely more on government funding sources. Effective care coordination for these inpatients might improve outcomes while reducing taxpayer burden.

## Background

Veterans who are enrolled in the VA healthcare system often use private sector medical services instead of or in addition to VA care, and several factors have been associated with whether VA or non-VA care is used. For example, enrollees who rely more on VA care tend to be sicker [1] and poorer [2]. They also tend to have been assigned higher “priority” for VA care [3], which a) is based on income and the extent to which injuries incurred during military service, or other catastrophic disabilities, impair functioning and the capacity for gainful employment, and b) determines whether veterans are eligible to receive certain services, reimbursed for driving to VA care, or required to make co-payments. Greater reliance on non-VA care, on the other hand, has been associated with Medicare or commercial health insurance coverage [4], which is widespread among veterans – among active VA patients surveyed in 2007, for example, 79 % had some other type of health insurance [5]. Additionally, how much veterans rely on VA versus non-VA care is influenced by the distances/times they must travel to reach service sites [3, 6–8]. VA hospitals typically serve very large regions, and for many enrollees, the nearest one is much farther away than the nearest non-VA hospital; veterans who obtain primary care services at VA community based outpatient clinics, which usually are located far from their parent hospitals, rely on non-VA hospitals for most of their inpatient care [9]. Diagnosis also is a factor – for example, VA enrollees younger than 65 rely more on non-VA care for most aggregated condition categories, with the exception of substance abuse, mental health disorders, or eye problems, for which reliance on VA care is greater [3].

Many VA-enrolled veterans are “dual users” who get both VA and non-VA medical care close in time. For these patients, the risks of poorly coordinated, fragmented care are likely to be greater than for those using a single healthcare system [10, 11]. About 80 % of veterans dually eligible for VA and Medicare services use both; among those younger than 65 years (therefore disabled), 58 % are dual users [3]. Compared to other VA patients, dual users of VA and Medicare services are more likely to a) be Caucasian, older, or married, b) have more education, higher incomes, lower priority for VA services, or higher levels of illness burden, and c) live farther from VA hospitals or in urban areas with more healthcare resources [3, 8, 11–15]. Studies of veterans’ use of Medicare, however, do not yield information on how most working age VA enrollees use non-VA healthcare that may be paid for by commercial insurance or other payers. Yet surveys of younger veterans’ non-VA healthcare use have revealed similar associations with demographics, income, disability/illness, and distance to care. Moreover, VA enrollees younger than 65 years are more likely to report being dual users if they have private insurance, though less likely to be dual users than older patients who have Medicare [16–20].

Patients with multiple hospitalizations, especially those with admissions close in time, are presumably among the sickest and most costly of patients, and their needs should be well understood for optimal resource use. Those who use both VA and non-VA inpatient care may be a very selective subset of dual users, whose characteristics differ from those described above. These dual users probably use different hospitals for their particular expertise and capacity (e.g., VA for mental health or rehabilitation, non-VA for high-technology surgery) and they likely have more hospitalizations overall, for a wider array of problems, both medical and mental. They may be at particular risk for problems that can arise from the potential discontinuities in, and possibly conflicting or duplicative, care obtained from multiple unintegrated providers. In fact, dual use of inpatient services has been associated with increased risk of mortality [11, 21]. Information about the characteristics and needs of multiply-hospitalized patients may help VA policymakers in allocating resources to better manage patients who use more than one system of care.

To estimate the extent to which VA-enrolled veterans have multiple hospitalizations within, say, a single calendar year, in either VA or non-VA hospitals, we analyzed a unique set of administrative hospital discharge data. Using non-VA discharge data acquired from agencies in seven states, merged with VA discharge data, we analyzed all VA and non-VA hospitalizations undergone in 2007 by VA-enrolled veterans of any age. Patients who had only one hospitalization (either VA or non-VA) in that year were distinguished from those who had more than one, and among patients with multiple hospitalizations, those who used only VA hospitals, only non-VA hospitals, or both types of hospital (dual users) were compared. Our objective was to determine whether multiply admitted patients, particularly dual users of both VA and non-VA hospitalizations, have unique characteristics that policymakers should consider in resource allocation or for designing targeted care coordination efforts. We anticipated that dual users of inpatient care in different age ranges would have distinctive demographics, diagnoses, and insurance use for non-VA care.

## Method

The study population included all veterans who in 2007 were currently enrolled in VA healthcare and living in Arizona, Iowa, Louisiana, Florida, South Carolina, Pennsylvania, or New York. We acquired administrative discharge data for all inpatient admissions to VA or non-VA hospitals that these enrollees had from 2004 through 2007. The agencies listed in the Acknowledgements as sources for the data gave their permission to use the information. In each state, a government agency or hospital association routinely collected administrative discharge data for all hospitalizations occurring in private sector hospitals within the state; data managers reported that all private sector hospitals were expected to report all admissions, and they believed that compliance rates were very high. These states also collected the patient identifiers (SSN, date of birth, and gender) needed to match admissions to individuals in our enrollment lists. VA’s National Data Systems (NDS) generated random unique ID numbers (UIDs) for all enrollees in these states and extracted discharge data for any VA hospitalizations they had. NDS also provided their personal identifiers and UIDs to the states’ managers of non-VA hospitalization data, who used them to search for exactly matching records in their databases. Matching records were returned without identifiers but with UIDs to enable the aggregation of each enrollee’s VA and non-VA hospitalizations, if applicable. Consequently, the data include any non-VA hospitalizations that VA enrollees had in their home states and any VA admissions they had anywhere. Their non-VA utilization may be under-represented if they had out-of-state hospitalizations, but all their VA hospitalizations are included (more than 95 % of which were in-state). This study was approved by Dartmouth College’s Committee for the Protection of Human Subjects (#20826).

Among the data elements for each hospitalization were the principal diagnosis, secondary diagnoses, diagnosis-related group (DRG), admission and discharge dates, age, gender, priority level for VA care, ZIP code of the patient’s residence, and the primary payer (for non-VA hospitalizations). In some non-VA datasets, principal diagnosis was not clearly identified; if not, we assumed that the first listed diagnosis was the principal diagnosis, which, though likely, may not always be true for admissions with multiple diagnoses. Principal diagnoses were assigned to major ICD-9-CM categories, and all diagnoses and DRGs were submitted to the Agency for Healthcare Research and Quality’s publicly available co-morbidity detection software [22] designed to search administrative discharge data for 30 physical or psychiatric co-morbid diagnoses that have been identified as important predictors of treatment outcomes [23]. For each hospitalization we counted the co-morbidities representing physical and mental disorders. We did not acquire patients’ Medicare enrollment status, which was generally unavailable in the non-VA data. However, non-VA hospitalization data did include the expected primary payer for the admission. Across the seven states we were able to categorize primary payers as Medicare, commercial insurance, or any other, which in various states might include Medicaid, TRICARE, other non-VA government sources, workmen’s compensation, charity, or self-pay (but typically were not broken out separately). For six states (not Florida), Medicaid also was a separate category.

We applied a published SAS routine [24] to access Google Maps for estimated driving times from the geographic centroid of each patient’s ZIP code to those of all VA hospitals within a 300-mile radius of that code, and selected the shortest travel time as a measure of proximity to VA acute care. We repeated this process to select the nearest non-VA hospital, using a comprehensive list drawn from Medicare’s Hospital Compare website [25]. Each patient’s residence was assigned to a Rural – Urban Commuting Area (RUCA) category [26, 27] by applying a publicly available ZIP code approximation to implement Categorization A [28], which defines four categories: Urban, Large Rural Town, Small Rural Town, and Isolated Small Rural Town. We also used enrollees’ ZIP codes to estimate household incomes based on Census Bureau ZIP code level median values for 1999 [29].

We limited data analyses to include only enrollees who were hospitalized (in either VA or non-VA hospitals) during calendar year 2007. We found 319,073 such individuals, most of whom had only one admission during the year; those who had more than one hospitalization in 2007 included 35,284 enrollees younger than 65 years (32.3 % of that age group) and 80,409 enrollees 65 years or older (38.3 % of that age group). All subsequent analyses included only the enrollees with multiple hospitalizations in 2007, for a total of 115,693 unique patients. We separated them into those who used only VA hospitals, those who used only non-VA hospitals, and those who used either at least once (dual users). Within each age range (younger or older than 65), we compared groups on available demographics, geographic variables, characteristics of their hospitalizations in 2007, payers for non-VA admissions, and numbers of hospitalizations during the three previous years, 2004–2006, using chi-square analyses or analysis of variance with general linear models. Because the large sample size yielded highly significant effects for small group differences, strength of association between nominal variables was assessed using Cramer’s V statistic, which can range from 0 (no association) to 1 (identical variables). We also applied multinomial logistic regression to predict group membership from demographic, geographic, and diagnostic variables, with patients who used only VA hospitals as the reference group for dual users and non-VA only users. All analyses were conducted using SAS version 9.2 (SAS Institute, Cary, NC).

## Results and discussion

The characteristics and hospital use of 115,693 patients who were hospitalized more than once in 2007 are summarized in Table 1; within each age range, dual users are compared to patients who used only one type of hospital, VA or non-VA. Due to large Ns, all tests of group differences within age ranges were significant at *p* < .001 or better, though effect sizes may be small; for categorical variables, Cramer’s V is included to show the strength of relationship to group membership. More than four-fifths of elderly patients, and one-half of those younger than 65 years, used non-VA hospitals exclusively. The remainder in each age range included a slightly higher proportion of patients who used only VA hospitals than of dual users.

**Table 1:** Characteristics and hospital use of 115,693 VA-enrolled veterans who were hospitalized more than once in 2007, by age range and type(s) of hospital used (VA only, non-VA only, or both, i.e., dual use)

Age Range:		Younger than 65 Years			65 Years or Older		
Hospital Use:		VA Only	Dual Users	Non-VA Only	VA Only	Dual Users	Non-VA Only
Patient Characteristics: N (% of age group)		9391 (26.6 %)	8354 (23.7 %)	17539 (49.7 %)	8129 (10.1 %)	6768 (8.4 %)	65512 (81.5 %)
Mean Age in 2007 (SD)		54.4 (8.0)	54.3 (8.0)	55.3 (7.9)	76.6 (7.2)	77.0 (7.1)	78.8 (6.7)
% Female		5.9 %	5.5 %	5.9 %	2.5 %	2.6 %	2.1 %
		Cramer’s V = 0.01			Cramer’s V = 0.01		
ZIP Code-based Median Household Income: Mean (SD)		$34673 (13947)	$35544 (12886)	$38001 (14140)	$36235 (14208)	$37163 (13640)	$41931 (16204)
% in VA Priority Categories: 1 (Highest Priority)		32.7 %	32.4 %	23.0 %	20.5 %	20.9 %	9.3 %
2		5.4 %	5.3 %	6.6 %	4.8 %	4.4 %	4.7 %
3		9.1 %	8.4 %	11.6 %	8.7 %	8.3 %	10.1 %
4		4.7 %	5.1 %	2.9 %	8.9 %	10.0 %	5.2 %
5		39.0 %	39.6 %	27.1 %	44.0 %	40.1 %	19.9 %
6		1.8 %	1.7 %	5.7 %	0.4 %	0.4 %	0.8 %
7		1.0 %	1.0 %	1.5 %	1.9 %	2.1 %	3.2 %
8 (Lowest Priority)		6.3 %	6.7 %	21.5 %	10.8 %	13.9 %	46.9 %
		Cramer’s V = 0.20			Cramer’s V = 0.23		
% Living in RUCA Categories: Urban		83.8 %	83.9 %	82.0 %	82.9 %	80.8 %	80.6 %
Large Rural Town		8.5 %	9.2 %	10.8 %	8.3 %	10.1 %	11.2 %
Small Rural Town		4.5 %	4.2 %	4.4 %	4.7 %	5.2 %	4.9 %
Isolated Small Rural Town		3.1 %	2.7 %	2.8 %	4.1 %	3.9 %	3.4 %
		Cramer’s V = 0.02			Cramer’s V = 0.02		
Minutes of Driving Time to Nearest Hospital:	Nearest VA Mean (SD)	40.0	51.0	60.9	36.2	45.9	59.0
		(37.4)	(78.0)	(45.4)	(34.0)	(37.6)	(115.0)
	Nearest non-VA Mean (SD)	12.4	12.0	12.7	13.8	13.1	12.3
		(13.1)	(11.8)	(12.2)	(14.2)	(12.9)	(11.5)
Use of Inpatient Care in 2007:							
Number of Hospitalizations per Person:	Any (VA or non-VA) Mean (SD)	2.7	3.8	2.9	2.8	3.5	2.8
		(1.3)	(2.8)	(1.8)	(1.3)	(2.0)	(1.4)
	VA Mean (SD)	2.7	1.9	0.0	2.8	1.7	0.0
		(1.3)	(1.6)	(−−)	(1.3)	(1.2)	(−−)
	non-VA Mean (SD)	0.0	1.9	2.9	0.0	1.8	2.8
		(−−)	(2.0)	(1.8)	(−−)	(1.6)	(1.4)
Days of Stay per Hospitalization:	Any (VA or non-VA) Mean (SD)	5.5	5.2	3.9	5.5	5.2	3.9
		(8.7)	(8.5)	(5.4)	(8.4)	(6.7)	(4.7)
	VA Mean (SD)	5.5	6.1	0.0	5.5	5.9	0.0
		(8.7)	(14.8)	(−−)	(8.4)	(10.9)	(−−)
	non-VA Mean (SD)	0.0	4.3	3.9	0.0	4.5	3.9
		(−−)	(6.7)	(5.4)	(−−)	(5.8)	(4.7)
Number of Co-morbid (Elixhauser) Diagnoses:	Physical Mean (SD)	1.9	1.6	1.4	2.6	2.3	1.9
		(1.3)	(1.1)	(1.3)	(1.3)	(1.2)	(1.3)
	Mental Mean (SD)	0.6	0.6	0.3	0.1	0.2	0.1
		(0.8)	(0.6)	(0.5)	(0.3)	(0,3)	(0.2)
% of Individuals With ≥ 1 Admission in 2007 for a Principal Diagnosis in the Category of: Circulatory System Diseases		26.2 %	33.0 %	40.7 %	44.1 %	52.1 %	53.8 %
		Cramer’s V = 0.13			Cramer’s V = 0.06		
Mental Disorders		31.4 %	38.1 %	15.0 %	0.2 %	0.2 %	0.0 %
		Cramer’s V = 0.23			Cramer’s V = 0.03		
Symptoms, Signs, & Ill-defined Conditions		16.8 %	22.1 %	16.1 %	20.8 %	23.0 %	14.5 %
		Cramer’s V = 0.06			Cramer’s V = 0.08		
Injury & Poisoning		12.7 %	20.3 %	20.9 %	13.6 %	18.0 %	18.3 %
		Cramer’s V = 0.09			Cramer’s V = 0.04		
Diseases of Respiratory System		11.4 %	17.0 %	15.9 %	22.6 %	27.3 %	21.9 %
		Cramer’s V = 0.06			Cramer’s V = 0.04		
Diseases of Digestive System		17.0 %	18.9 %	18.2 %	17.3 %	18.2 %	17.9 %
		Cramer’s V = 0.02			Cramer’s V = 0.01		
Diseases of Genitourinary System		9.2 %	8.9 %	7.8 %	16.7 %	18.0 %	13.9 %
		Cramer’s V = 0.02			Cramer’s V = 0.04		
% of Individuals Hospitalized for Diagnoses in Only One, Two, or More than Two Major Categories:	One	43.0 %	28.9 %	35.7 %	29.8 %	21.1 %	26.9 %
	Two	45.6 %	46.5 %	49.3 %	53.7 %	48.0 %	53.8 %
	More than Two	11.4 %	24.6 %	15.0 %	16.5 %	30.9 %	19.3 %
		Cramer’s V = 0.10			Cramer’s V = 0.06		
Hospitalizations for Circulatory System Diseases:	Mean (SD) % in VA Hospitals	0.4	0.7	0.7	0.7	1.0	0.9
		(0.8)	(1.3)	(1.1)	(1.0)	(1.4)	(1.1)
		100 %	46.9 %	0 %	100 %	47.3 %	0 %
Hospitalizations for Mental Disorders:	Mean (SD) % in VA Hospitals	0.7	1.0	0.3	0.0	0.0	0.0
		(1.2)	(2.0)	(1.1)	(0.1)	(0.1)	--
		100 %	57.9 %	0 %	100 %	66.7 %	0 %
% of Individuals Hospitalized for Mental Disorders and also Hospitalized for non-Mental Diagnoses		13.1 %	26.0 %	9.3 %	0.2 %	0.2 %	0.0 %
		Cramer’s V = 0.19			Cramer’s V = 0.03		
Prior Hospital Use, in 2004 – 2006:							
% with Any Prior Hospitalizations		64.6 %	68.9 %	63.0 %	67.9 %	70.3 %	67.4 %
		Cramer’s V = 0.05			Cramer’s V = 0.02		
% with Any Prior VA Hospitalizations		57.2 %	50.3 %	11.9 %	60.7 %	47.8 %	4.4 %
		Cramer’s V = 0.32			Cramer’s V = 0.41		
% with Any Prior non-VA Hospitalizations		25.2 %	49.3 %	59.7 %	23.6 %	47.9 %	66.1 %
		Cramer’s V = 0.21			Cramer’s V = 0.20		
Mean Prior VA Hospitalizations (SD)		2.0	1.7	0.3	1.9	1.3	0.1
		(3.0)	(3.0)	(1.0)	(2.6)	(2.3)	(0.5)
Mean Prior non-VA Hospitalizations (SD)		0.5	1.9	2.4	0.5	1.4	2.1
		(1.4)	(4.3)	(4.2)	(1.2)	(2.7)	(1.8)

Due to large Ns, all (within age group) ANOVAs and *χ* ^2^ analyses were significant at *p* < .001, though variance explained could be low. Cramer’s V shows strength of relationship for categorical variables (very strong: 0.25+; strong: 0.15–0.25; weak – moderate: 0.06–0.15; negligible: 0.01–0.05)

In either age range, dual users differed very little from other groups in sex distributions, and they closely resembled VA-only users in mean age or income and in distributions among VA priority categories. Non-VA-only users, on the other hand, were slightly older and had higher incomes, on average, and were less likely to be in VA priority categories 1 (highest service-related disabilities) or 5 (impoverished) and more likely to be in category 8 (lowest priority). Groups differed little with respect to residence in RUCA categories, but on average, dual users lived farther from VA hospitals than VA-only users and closer than non-VA-only users. Though analyses of travel times to non-VA hospitals also yielded significant group effects, means were low and differed little. Briefly, dual users were similar to VA-only users in age, income and VA priority, but lived farther from VA hospitals.

Dual users’ hospitalizations were split evenly between VA and non-VA, and they averaged more admissions overall than other groups, though fewer within each system than patients who used that system exclusively. Dual users also averaged longer stays than other patients, in either VA or non-VA hospitals. They averaged slightly fewer co-morbid diagnoses across all hospitalizations than VA-only users did, but this disparity could be due to different practices in VA and non-VA hospitals for coding secondary diagnoses. Dual users also were more likely to be hospitalized for principal diagnoses in several common categories: They were more likely than VA-only patients to be hospitalized for diseases of the circulatory system (though less likely than non-VA-only patients). Among younger patients, dual users were most likely to be hospitalized for mental disorders, more so than other VA inpatients; psychiatric admissions were considerably less common among non-VA patients, and very rare among elderly patients. In either age range, dual users were more likely than other patients to be admitted for symptoms, signs, and ill-defined conditions, and they were similar to non-VA-only users in being more likely than VA-only users to have admissions for injury and poisoning. Though associations with patient group (Cramer’s V) were weak, dual users were consistently among the most likely to have admissions for respiratory, digestive, or genitourinary diseases. As they tended to have more admissions than other patients, dual users also were more likely to be hospitalized for principal diagnoses in three or more major categories. They averaged the most admissions for circulatory diseases and mental disorders, and they obtained roughly half of those for either category in VA hospitals. Among patients younger than 65 years, dual users were considerably more likely to have admissions for both mental disorders and non-mental diagnoses. Briefly, dual users had more hospitalizations, for more reasons.

Compared to single system users who had multiple hospitalizations in 2007, dual users were about as likely to have had prior admissions during the preceding three years; furthermore, they were only somewhat less likely than VA-only users to have had prior VA admissions, and somewhat less likely than non-VA-only users to have had prior non-VA admissions. Compared to these other patients, dual users averaged more prior hospitalizations overall, which were split about equally between VA and non-VA. Dual users in 2007, therefore, were likely to have had histories of dual hospital use.

We then sought to determine which variables best predicted group membership (VA, non-VA, or dual use) by applying multinomial logistic regressions (one for each age range) with these predictor variables: sex, income, VA priority, RUCA residence category, driving times, prior hospitalization, and dummy variables for whether the patient had any 2007 or prior admissions for principal diagnoses in each of several general categories. We used VA-only patients as the referent group, and generated odds ratios to assess whether each predictor affected the likelihood that a patient was a dual or non-VA-only user instead; odds ratios are presented in Table 2.

**Table 2:** Odds ratios from multinomial logistic regressions (one for each age range) to predict type(s) of hospitals used by patients with multiple hospitalizations in 2007

Age Range:		Younger than 65 Years		65 Years or Older	
Hospital Use (Referent = “VA Only”):		Dual Users	Non-VA Only	Dual Users	Non-VA Only
Sex: Referent = Male	Female	0.98	1.18	0.99	0.84
ZIP Code estimated 1999 household income: Referent = Less than $33500	$33500-$42500	1.07	1.25**	1.09	1.54**
	> $42500	1.14*	1.61**	1.23**	2.30**
Priority for VA care: Referent = Level 1 (Highest priority)	Level 2	1.04	1.54**	0.96	1.99**
	Level 3	0.93	1.65**	0.97	2.28**
	Level 4	1.02	0.98	1.12	1.62**
	Level 5	1.02	1.05	0.95	1.04
	Level 6	1.00	3.16**	0.98	3.12**
	Level 7	1.06	1.75*	1.02	2.77**
	Level 8	1.12	3.64**	1.28*	6.64**
RUCA category of patient residence: Referent = Urban	Large Rural	0.90	0.95	1.03	1.07
	Small Rural	0.77*	0.68**	0.92	0.95
	Isolated Rural	0.74*	0.63**	0.80	0.68**
Driving time to nearest VA hospital: Referent = Less than 60 min	More than 60 min	1.88**	2.72**	1.99**	3.77**
Driving time to nearest non-VA hospital: Referent = Less than 15 min	More than 15 min	0.91	0.91	0.83**	0.74**
VA hospitalizations during 2004–2006: Referent = None	Any	0.52**	0.10**	0.49**	0.04**
Non-VA hospitalizations during 2004–2006: Referent = None	Any	2.51**	5.97**	2.62**	7.15**
Having any hospitalizations in 2004–2007 for diseases in each of several major diagnostic categories: Referent = None	Circulatory System	1.35**	1.56**	1.37**	1.48**
	Digestive System	1.08	1.08	1.03	1.14*
	Respiratory System	1.37**	1.32**	1.21**	1.12*
	Genitourinary System	0.91	0.86*	1.04	0.91
	Mental Disorders	1.60**	0.69**	1.15	0.52*
	Symptoms, Signs, Ill-Defined Conds	1.24**	0.97	1.04	0.76**
	Injury & Poisoning	1.44**	1.61**	1.31**	1.38**
	Musculoskeletal & Connective	1.04	1.24**	1.14	1.43**
	Neoplasms	0.83*	0.69**	0.87*	0.70**
	Other Diagnoses	1.23**	1.13*	1.38**	1.18**

* → *p* < .01; ** → *p* < .0001

In either age range, sex did not significantly change the odds of group membership, but income did. Compared to patients in the lowest third of incomes, others were substantially more likely to use non-VA hospitals exclusively; those in the highest third of incomes also were more likely to be dual users. Compared to patients with the highest priority for VA care (category 1), those in categories 2, 3, 6, 7, and 8 (and elderly patients in category 4) were more likely to use only non-VA hospitals; patients in categories 6 and 8 were especially less likely than category 1 patients to use VA hospitals at all. VA priority did not change the odds of being a dual rather than a VA-only user, except among elderly patients with the lowest-priority.

Rural residence seems to have affected hospital use more for younger than elderly patients: Younger patients living in small or isolated small rural towns were less likely than urban residents to use non-VA hospitals, either as dual or exclusive users; elderly patients in isolated small rural towns also were less likely to use non-VA hospitals exclusively. Travel times, on the other hand, seem to have affected hospital use more among elderly patients: In either age range, the one-third of patients who lived more than an hour from the nearest VA hospital were almost twice as likely to be dual users. They were nearly 3 times as likely to be non-VA-only users if they were younger than 65, and nearly 4 times as likely if elderly. Elderly patients also were less likely to use non-VA hospitals, either as dual or exclusive users, if they belonged to the roughly one-third who lived more than 15 min from the nearest non-VA hospital.

Prior hospital use was among the strongest predictors of hospital use in 2007. Patients who had any VA admission(s) during the three preceding years were considerably more likely to use only VA hospitals in 2007 than to be dual users in that year; they were even less likely to be exclusively non-VA users in 2007. Conversely, patients with any prior non-VA admissions were considerably more likely to use only non-VA hospitals in 2007 than to be dual users or VA-only users in that year.

Regarding principal diagnoses, in either age range patients who had any admission, in 2007 or during the prior three years, for diseases of the circulatory or respiratory systems, injury and poisoning, or other, less common diagnostic categories, were more likely to use non-VA hospitals, either as exclusive or dual users, than VA hospitals only. On the other hand, patients were more likely to use VA hospitals exclusively if they had any admission for neoplasms. Patients also were more likely to use non-VA hospitals exclusively if they had any admissions for diseases of the musculoskeletal system and connective tissue, but less likely to do so if they had any admissions for mental disorders. Patients younger than 65 were more likely to be dual users if they had any admissions for mental disorders or for symptoms, signs, and ill-defined conditions, and they were less likely to use non-VA hospitals exclusively if they had any admissions for genitourinary diseases. Elderly patients also were more likely to use only non-VA hospitals if they had any admissions for digestive system disease, but less likely to do so if they had admissions for symptoms, signs, and ill-defined conditions.

In other words, circulatory diseases, respiratory diseases, injury and poisoning, less common diseases, diseases of the musculoskeletal system and connective tissue, and digestive diseases appear to increase the likelihood of using non-VA hospitals, whereas mental disorders, neoplasms, and symptoms, signs, and ill-defined conditions are more likely to be treated in VA hospitals. One reason may be that the latter diagnostic categories are associated with longer hospital stays, which non-VA hospitals have greater incentives to keep short. Compared to patients who use only VA hospitals, dual users are more likely to have admissions for mental disorders, circulatory diseases, respiratory diseases, injury and poisoning, less common diagnoses, and symptoms, signs, and ill-defined conditions, but less likely to have admissions for neoplasms.

Table 3 shows the percentages of non-VA hospitalizations with payers in each category, for patients younger or older than 65 years. Older patients relied less on Medicare and more on payers other than commercial insurance if they were dual users. Younger patients, on the other hand, relied less on commercial insurance and more on payers other than Medicare if they were dual users. Medicaid was not used frequently, though patients younger than 65 used it substantially more often than the elderly.

**Table 3:** Payers of non-VA hospitalizations in 2007, by patient type and age range

Patient Age Range:	Payer:	Dual Users	non-VA Only Users
Younger than 65 Years Old	Medicare	30.5 %	33.4 %
	Private Insurance	21.4 %	39.1 %
	All Other Payers	48.1 %	27.5 %
		Cramer’s V = 0.20	
65 Years or Older	Medicare	74.8 %	87.7 %
	Private Insurance	12.1 %	9.8 %
	All Other Payers	13.1 %	2.5 %
		Cramer’s V = 0.15	
For Six States (Not Florida), “All Other Payers” Could Be Separated Into:			
Younger than 65 Years Old	Medicaid	12.1 %	9.4 %
	Other Payers	36.0 %	18.1 %
65 Years or Older	Medicaid	0.9 %	0.2 %
	Other Payers	12.1 %	2.3 %

“All Other Payers” might include Medicaid, TRICARE, other non-VA government sources, workmen’s compensation, charity, self-pay, or any other source not covered by Medicare or private insurance. Aside from Medicaid, each of these other payers was broken out separately in only one or a few of the states. For six states (not Florida), Medicaid also was a separate category

In summary, about one-third of veterans enrolled in the VA healthcare system who were hospitalized in 2007 had more than one hospitalization during that year. Of these multiply hospitalized patients, one of every twelve elderly patients was a dual user who had at least one VA and one non-VA admission; among younger patients, one of every four was a dual user. The age discrepancy reflects the fact that most elderly patients used non-VA hospitals exclusively, due to their Medicare coverage; numbers of dual users, however, were substantial in either age group. Relying on VA and non-VA hospitals about equally, dual users averaged more hospitalizations and longer hospital stays in 2007, as well as more admissions in prior years than other multiply hospitalized patients.

Per our study objectives, we found that dual users were similar to VA-only users with respect to sex, age, income, and priority for VA care, but they lived farther from the nearest VA hospital and were less likely to have used VA hospitals in past years (though more likely to have used non-VA hospitals). Consequently, greater distances to VA care might have induced some dual users to get some of their inpatient care in non-VA hospitals. Dual users also were more likely than VA-only users to have been hospitalized for principal diagnoses in a variety of categories, including circulatory diseases, injury and poisoning, respiratory diseases, and other, less common categories, and were more likely to have admissions for multiple diagnostic categories. Dual users younger than 65 had the most admissions for mental disorders, and were more likely to have admissions for mental disorders as well as for physical conditions.

Dual users also relied more on funding sources other than Medicare or private insurance to pay for their non-VA care. Since private health insurance plans often impose benefit limits on mental health stays, patients with greater mental health needs may use both non-VA and VA care, particularly given the VA’s capacity and recognized expertise with combat-related PTSD and other psychiatric conditions. Where prior studies have found that VA enrollees who were dual users were more likely to have commercial insurance, the dual users in this study were much less likely than other patients to have their non-VA hospitalizations reimbursed by insurance. The discrepancy is likely due to our having only inpatient and no outpatient information for these patients – our definition of “dual use” is necessarily narrow, based on an individual having both VA and non-VA hospitalizations, and represents only a subset of dual users as other studies have defined them. There are likely to have been many patients that we designated as exclusive VA or non-VA users who in fact used outpatient or other services in both VA and non-VA systems of care. Though our “dual users” are likely sicker and more disabled than other veterans who use both VA and non-VA outpatient services, they probably under-represent the numbers of patients at risk for the negative consequences of disjointed care due to dual use.

Other limitations warrant consideration when interpreting results: Data were acquired from only seven states, unevenly distributed geographically, with a bias to the East. In identifying data sources for this study we found that only these states a) collected hospital discharge data from all in-state non-VA hospitals that included the personal identifiers needed to find data for VA enrollees, and b) were willing to provide us the data, including a random number identifier for matching to VA treatment data. Therefore these states might not be representative of other states. On the other hand, our data include all private sector hospitalizations (not just Medicare admissions) that VA enrollees of any age had, and represent a unique aggregation of data from numerous hospitals, both VA and non-VA. Admittedly, the data come from administrative hospital discharge datasets created by each state’s responsible government agency or hospital association, each with unique coding formats and practices. Administrative data may be susceptible to a variety of undetectable coding inaccuracies; moreover, because non-VA providers’ reimbursements relate directly to services rendered while VA providers’ do not, diagnostic coding incentives likely differ substantially. Our VA treatment data also are more complete, as they include all the enrollees’ VA hospitalizations, even in other states (though 95 % were in-state and another 3-4 % were in adjacent states); our non-VA data, on the other hand, include only those hospitalizations that occurred in the states in which enrollees lived. Furthermore, some non-VA data may be missing if there were reporting failures or if inaccurate identifiers prevented correct matches when each state extracted its data. Collection of the non-VA data was mandatory, and state data managers believed that non-VA hospitals reported admissions very reliably (estimated to be > 90 %). Across the states, non-VA hospitalization rates per VA enrollee were usually higher and varied substantially more than VA rates; though this variability might reflect reporting failures to an unknown extent, estimates based on the seven states combined should be highly reliable and representative of both VA and non-VA use.

Another limitation might be our use of data that are almost a decade old. Acquisition of these data was a long and difficult process that is unlikely to be replicated, so they provide a very unique source of information on veterans’ dual use, which we believe remains very relevant today. For the sake of efficiency, hospitals, VA or non-VA, tend to keep their beds full (or nearly so); numbers of hospital beds also tend not to change dramatically within a few years. Consequently, reliance on VA versus non-VA inpatient care is not likely to fluctuate nearly as much as outpatient use might. The Affordable Care Act might increase the number of VA enrollees, and therefore increase utilization of VA healthcare, but it also will increase non-VA access for many enrolled veterans; its overall impact on VA reliance remains uncertain [30]. To the extent that the ACA expands coverage for mental disorders, non-VA psychiatric hospitalizations will likely increase. Given the constraints of hospital bed availability, however, we believe that our findings will generally be relevant for some time to come.

Our definition of dual use (i.e., admissions to both VA and non-VA hospitals in a single calendar year) is much narrower than other studies have employed; yet of all veterans who had a VA hospitalization in 2007, 19 % of those younger than 65 years and 21 % of elderly patients qualified. An implication is that one of every five patients admitted to VA hospitals also recently had or soon will have a non-VA hospitalization. Awareness of care that veterans receive outside one’s system could help both VA and non-VA clinicians to better manage treatment, particularly since these patients may be the sickest, with both medical and psychiatric issues. Non-elderly patients with serious psychiatric and medical (especially circulatory) conditions seem to be considerably more likely to be dual hospital users, so these difficult patients may be at greater risk for problems associated with uncoordinated care. Systematically collecting patient information about prior hospital use can be difficult, but a standard method for identifying a patient’s non-VA treatment providers might yield great benefits to patient management. Steps to make electronic exchange of health care information more efficient and automatic would also help to promote coordinated care for veterans. Dual users are more likely to have non-VA hospitalizations paid for by funding sources other than commercial insurance or Medicare, including Medicaid and other government programs such as TRICARE and worker’s compensation. To the extent that more intensive care coordination can reduce the high number of hospitalizations they have, total taxpayer burden will be reduced.

## Conclusion

People who are hospitalized two or more times in the same year presumably need intensive healthcare services to a much greater extent than other patients. We found that of all VA-enrolled veterans in seven states who were hospitalized in 2007, 36 % were hospitalized more than once that year. Of these multiply hospitalized patients, veterans who used both VA and non-VA hospitals were admitted for more diagnoses and longer stays than those who used one system only. Younger dual users also were more likely to be hospitalized for mental disorders and less likely to have their non-VA admissions paid for by private insurance. Hence dual users appear to include more complex patients who rely heavily on public funding for their healthcare.

Of all patients admitted to VA hospitals in 2007, one-fifth also had a non-VA hospitalization during that year. Ascertaining whether patients are using both VA and non-VA hospital care will promote more effective care coordination, follow-up and patient safety, which might reduce hospitalizations overall.

## Abbreviations

VA: Veterans Health Administration
NDS: National Data Systems
UID: Unique identification
SSN: Social Security Number
ICD-9-CM: International Classification of Diseases, 9th Revision, Clinical Modification
DRG: Diagnosis related group
SAS: Statistical Analysis System
RUCA: Rural–urban Commuting Areas
